# Biochemical Pathways of Sarcopenia and Their Modulation by Physical Exercise: A Narrative Review

**DOI:** 10.3389/fmed.2017.00167

**Published:** 2017-10-04

**Authors:** Mohammad Mosaferi Ziaaldini, Emanuele Marzetti, Anna Picca, Zsolt Murlasits

**Affiliations:** ^1^Sport Physiology Department, Ferdowsi University of Mashhad, Mashhad, Iran; ^2^Department of Geriatrics, Neurosciences and Orthopedics, Catholic University of the Sacred Heart, Rome, Italy; ^3^Sport Science Program, College of Arts and Sciences, Qatar University, Doha, Qatar

**Keywords:** muscle atrophy, physical activity, apoptosis, inflammation, mitochondria

## Abstract

Aging is a complex process characterized by progressive multisystem derangement predisposing individuals to increased risk of developing negative health outcomes. Sarcopenia is the age-related decline of muscle mass and function/strength and represents a highly prevalent correlate of aging. Several factors have been indicated to play a role in the onset and progression of sarcopenia; however, its pathophysiology is still unclear. Physical exercise is to date one of the few strategies able to improve muscle health in old age through multiple metabolic and transcriptional adaptations. Although the benefits of different exercise modalities on the function and structure of aged myocytes is acknowledged, the cellular and molecular mechanisms underlying such effects are not yet fully identified. Here, we briefly overview the current knowledge on the biochemical pathways associated with the onset and progression of sarcopenia. We subsequently describe the effects of exercise on relevant signaling pathways involved in sarcopenia pathophysiology.

## Introduction

Sarcopenia is the progressive loss of muscle mass and strength/function during aging and has been increasingly recognized as a relevant factor for the occurrence of negative health outcomes in late life (e.g., falls, morbidity, disability, loss of independence, and mortality) (1). Indeed, sarcopenia is endorsed as a reliable biomarker allowing for the discrimination, at a clinical level, of biological from chronological age (2).

Despite the growing interest surrounding muscle aging, several intrinsic limitations still exist impeding its appreciation as a paradigm to study the aging process. First of all, the lack of a univocal operational definition of sarcopenia beside an unbiased method for the assessment of muscle mass and function hampers the incorporation of sarcopenia in everyday clinical practice (1). In addition to this, the incomplete knowledge of the pathophysiology of this condition halts the identification of targets that could be exploited for the development of intervention strategies (3).

**Figure 1:**
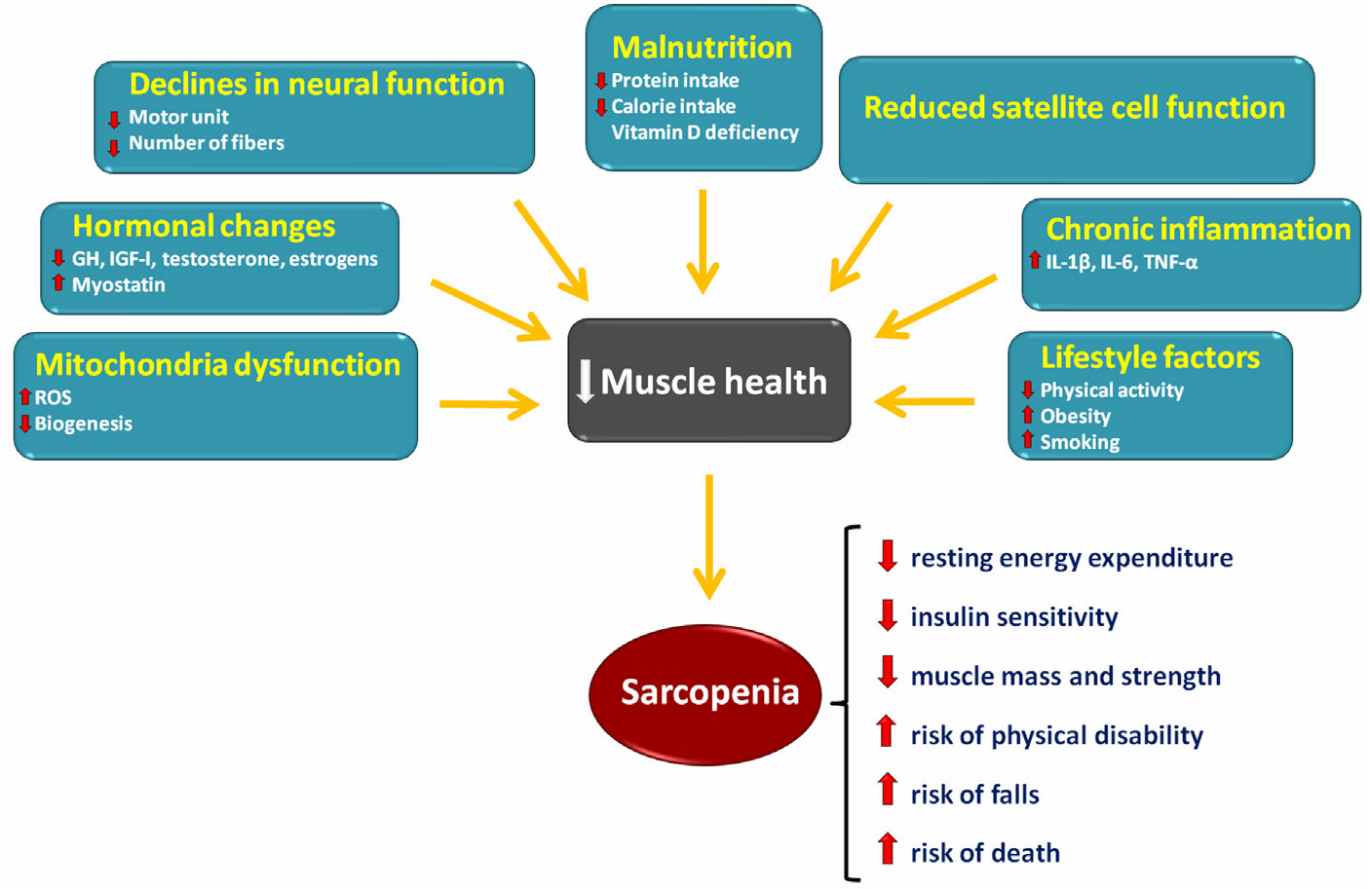
Schematic representation of the main factors involved in the onset and progression of sarcopenia and its consequences.

Low levels of physical activity are among the most important factors involved in the development of sarcopenia (4, 5) (Figure 1). Resistance training (RT) is the exercise strategy usually recommended to counteract age-related muscle wasting (6). Recent studies have shown that aerobic exercise training is also able to attenuate the rate of sarcopenia development (7–9). However, the precise cellular mechanisms through which resistance and aerobic training act on sarcopenia pathophysiology have not yet been fully appreciated.

Here, we briefly overview the current knowledge on the pathways involved in the onset and progression of sarcopenia. Subsequently, we describe the most notable biochemical adaptations elicited in muscle by physical exercise with the aim of pinpointing relevant pathways potentially useful for drug development.

## Signaling Pathways Involved in Sarcopenia

**Figure 2:**
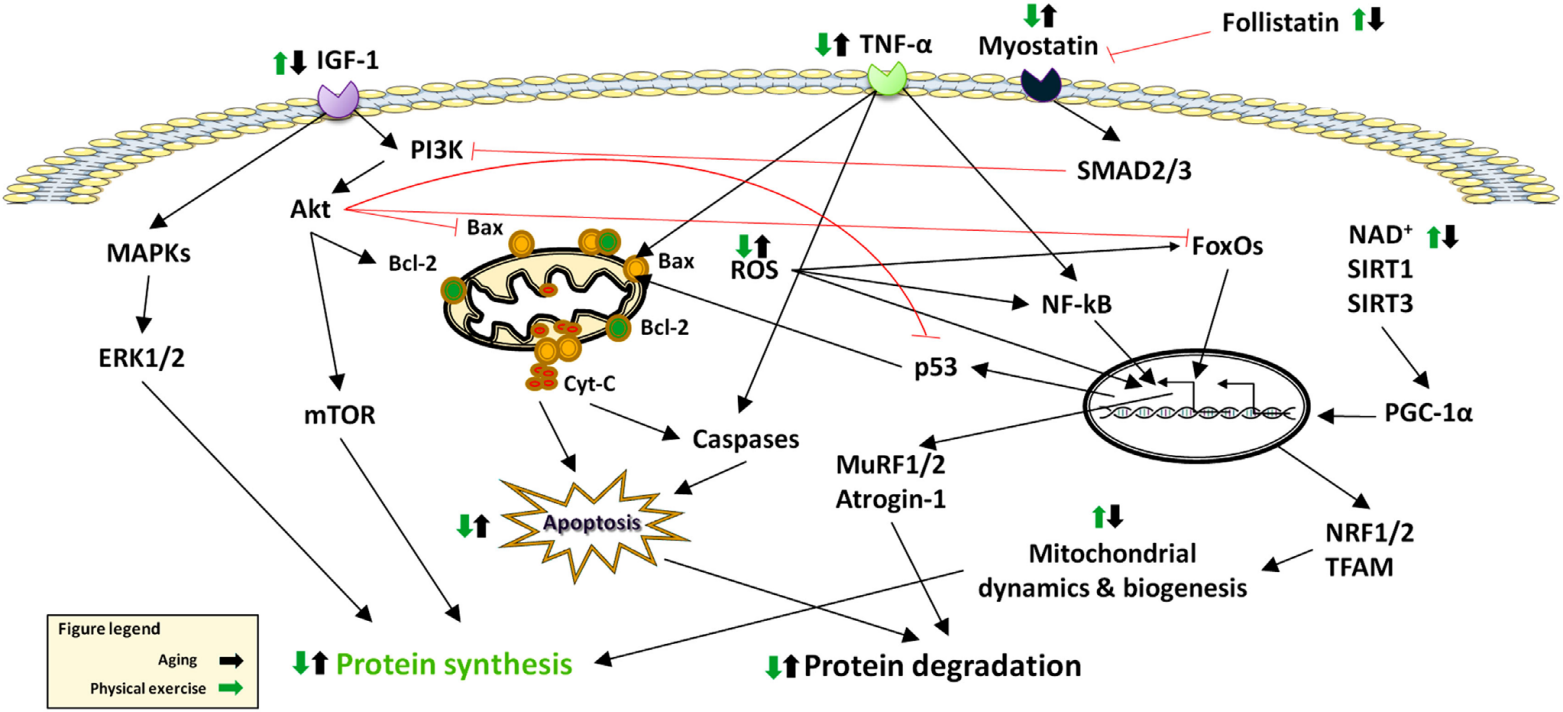
Effects of aging and physical exercise on signaling pathways altered in sarcopenia. Abbreviations: Cyt-C: cytochrome *C*; ERK: extracellular signal-regulated kinase; FoxO: Forkhead box O; IGF-1: Insulin-Like Growth Factor 1; MAPKS: Mitogen-Activated Protein Kinases; mTOR: mammalian target of rapamycin; MuRF: muscle RING-finger protein; NF-κB: nuclear factor κB; NRF: nuclear respiratory factor; PGC-1α: peroxisome proliferator-activated receptor-γ coactivator-1α; ROS: reactive oxygen species; SIRT: sirtuin; SMAD: small mother against decapentaplegic; TFAM: mitochondrial trascription factor A; TNF-α: tumor necrosis factor alpha.

The maintenance of skeletal muscle mass depends on the balance between anabolic and catabolic pathways (10) (Figure 1). The following sections summarize the current knowledge on major signaling pathways that are involved in sarcopenia (Figure 2).

### Insulin-Like Growth Factor 1 (IGF-1)/Akt/Mammalian Target of Rapamycin (mTOR)

Muscle size is under the control of the phosphatidylinositol-3-kinases (PI3K)/Akt pathway that is modulated by IGF-1 and insulin. These hormones stimulate protein synthesis and promote muscle hypertrophy by interacting with their respective tyrosine kinase receptors to phosphorylate the insulin receptor substrate 1 (IRS-1). As a result, PI3K/Akt is activated and stimulates mTOR. The latter eventually phosphorylates the 70-kDa ribosomal S6 protein kinase (S6K) and 4E-binding protein 1 (4E-BP1) (12), ultimately promoting protein synthesis.

Akt/protein kinase B is a Ser/Thr kinase that has been shown to be a critical signaling component in the regulation of cell metabolism, growth, and survival (13).

### Forkhead Box O (FoxO) Transcription Factors

FoxO transcription factors consist of a large family of proteins identified by a protected DNA-binding domain referred to as FoxO (14). FoxO family members involved in skeletal muscle physiology include FoxO1, FoxO3, and FoxO4 (15). FoxOs are predominantly located in the nucleus where they regulate the expression of a number of downstream signaling proteins. However, when FoxOs are phosphorylated, mainly by Akt, they are rerouted into the cytosol and therefore become unable to transcribe genes involved in muscle atrophy (16). Recent studies have provided evidence that FoxO1 suppresses the efficiency of anabolic pathways in muscle *via* increased expression and reduced phosphorylation of the translational repressor protein 4E-BP1 and impaired signaling *via* reductions in mTOR and regulatory-associated protein of mammalian target of rapamycin (RAPTOR) levels (14). Furthermore, elevated levels of myonuclear levels of FoxO1 have been found in muscle samples from older persons compared with younger counterparts (17).

### Transforming Growth Factor Beta (TGFβ)

Muscle regeneration is primarily modulated by members of the TGFβ superfamily, which are known to suppress myogenic differentiation (18). In particular, myostatin is one of the main signaling molecules that regulate muscle growth. Myostatin is produced by skeletal myocytes and negatively regulates muscle growth (15). The effects of myostatin are mediated by the transcription factors small mother against decapentaplegic (SMAD) 2 and 3, which also interfere with IGF1–Akt signaling. Myostatin has been reported to upregulate the ubiquitin ligases atrogin1 and muscle RING-finger protein-1 (MuRF1) *via* FoxO transcription factors. Indeed, myostatin administration has been shown to block the IGF1–PI3K–Akt pathway, thus activating FoxO1, allowing increased expression of atrogin-1. This connection between the two pathways is independent of nuclear factor κB (NF-κB) (19). In contrast, SMAD2/3 inhibition promotes muscle hypertrophy, which is partially dependent on mTOR signaling (20).

### NF-κB

NF-κB is a pleiotropic transcription factor involved in immune system modulation, inflammation, cell survival, and proliferation. NF-κB activity seems to directly regulate the expression of myogenic differentiation 1 protein (MyoD), a myogenic transcription factor, and likely other molecules, such as MuRF1, during atrophy. Reactive oxygen species and tumor necrosis factor alpha (TNF-α) both activate NF-κB (14). The binding of NF-κB to inhibitors of κB (IκB) is responsible for maintaining NF-κB in an inactive form in the cytosol. Seven isoforms of IκB exist in mammals (IκBα, IκBβ, IκBγ, IκBɛ, Bcl-3, p100, and p105), each possessing the ability to inhibit NF-κB. Upon certain stimuli, IκBα is phosphorylated by the IκB kinase in a step that targets IκBα for ubiquitination and subsequent proteolysis, thereby leaving NF-κB unbound. This process allows the unbound NF-κB to translocate to the nucleus where it can affect gene expression by binding NF-κB-target sequences located in the promoter region of specific genes (21).

### Mitogen-Activated Protein Kinases (MAPKs)

MAPKs are Ser/Thr kinases that transduce extracellular signals able to regulate a broad range of cellular processes. Indeed, in eukaryotic cells, the coordination of multiple MAPK pathways control gene expression, cell division, metabolism, motility, survival, apoptosis, and differentiation (22). The MAPK protein family is composed of four distinct signaling modules in skeletal muscle: (1) extracellular signal-regulated kinase (ERK) 1/2, (2) p38 MAPK, (3) c-Jun N-terminal kinases (JNKs), and (4) ERK5 or big MAPK. MAPKs are activated by cytokines, growth factors, and cellular stressors (23) and are stimulated by phosphorylation at regulatory tyrosine and threonine residues by upstream MAPK kinases. MAPK phosphatases are instead responsible for MAPK deactivation through dephosphorylation.

## Effects of Exercise Training on Muscle Pathophysiology

Sedentary lifestyle impacts muscle mass and strength as well as physical performance (24). Conversely, physical exercise, namely the body movements performed to maintain or improve components of physical fitness (25), is a powerful modulator of multiple processes involved in muscle hypertrophy and strengthening (13). Physical exercise is typically distinguished in endurance training (ET), which involves low-resistance work for protracted periods of time, and RT, characterized by more powerful movements of shorter duration (26). Both exercise regimens act on most signaling pathways involved in sarcopenia (27), including the IGF-1/Akt/mTOR axis (28–30), FoxOs (31, 32), NF-κB (33, 34), MAPKs (35, 36), mitochondrial quality control processes (37), and apoptosis (38–40). The following subsections summarize the specific effects of ET and RT on such pathways (Figure 2).

### Endurance Training

Aerobic exercise capacity decreases with advancing age, partly because of a decrease in the quantity and quality of muscle mitochondria (41, 42). On the other hand, ET improves maximal oxygen consumption (VO_2max_), mitochondrial density and activity, insulin sensitivity, and energy expenditure (43). Furthermore, ET reduces intramuscular fat accumulation and improves muscle function (44). The increase in citrate synthase (CS) activity in muscle fibers following ET supports the notion that this intervention improves mitochondrial mass (45). Numerous studies have investigated the effects of acute (13, 46–48) and chronic ET (49–63) on age-related muscle changes in both rodents and humans.

To investigate the acute effect of ET on skeletal muscle mitochondria in older persons, Bori et al. (48) studied the impact of a single bout of ET on the transcription of genes involved in mitochondrial biogenesis in sedentary versus physically active older adults. Their findings suggest that mitochondrial fission is impaired with age which could be involved in the age-associated decline in mitochondrial biogenesis-related gene expression in response to regular physical activity and exercise. These data also indicate that aging slightly affects the expression of mitochondrial biogenesis and quality control genes. Interestingly, old physically active participants showed similar levels of VO_2max_, mitochondrial density, CS activity, and cytochrome *c* oxidase (COX) of young sedentary individuals, emphasizing the impact of regular physical activity on muscle health (48).

In contrast to acute ET, chronic ET appears to have considerably greater effects. Konopka et al. (63) examined the influence of 12 weeks of progressive ET on markers of mitochondrial content in old women. Compared with basal levels, ET significantly increased the content of the peroxisome proliferator-activated receptor-γ coactivator-1α (PGC-1α) protein along with CS, β-hydroxylacyl CoA dehydrogenase, succinate dehydrogenase, and COX4. In addition to this, the expression of the mitochondrial fusion factors mitofusin (Mfn) 1 and 2 and fission protein 1 (Fis1) was increased by ET (63). In keeping with these findings, 12 weeks of ET were able to stimulate mitochondrial biogenesis and improve mitochondrial networking and the efficiency of mitochondrial energy transfer in old rats (60). ET also increased the content of COX4 and dynamin-related protein 1 (Drp1), but not that of Mfn1. Finally, ATP synthase activity, an indicator of mitochondrial energy production, was increased by ET (60). Upregulation of PGC-1α signaling is a major adaptation of skeletal muscle to ET (64). For instance, 12 weeks of ET were shown to increase PGC-1α content by 2.3-fold in old rats (61). This adaptation was associated with significant increases in mitochondrial trascription factor A (TFAM), cytochrome *c*, and mtDNA contents. Noticeably, as a response to ET, increased levels of upstream signaling mediators modulating PGC-1α activity, such as AMP-activated protein kinase (AMPK), p38MAPK, sirtuin 1 (SIRT1), and p-cAMP response element-binding protein (CREB) have been reported. These findings indicate that the age-associated decline in mitochondrial protein synthesis in skeletal muscle can be attenuated by ET (61). In this regard, 4 months of ET were found to increase the content of complexes III, IV, and V of the electron transport chain in muscles of older adults (62). Furthermore, a significant correlation was observed between TFAM and PGC-1α expression levels after 4 months of exercise intervention. However, no changes in expression levels of nuclear respiratory factor (NRF) 1 and 2 were detected in responses to ET (62).

Another mechanism through which ET positively impacts muscle aging involves the inhibition of myonuclear apoptosis (7, 14, 65). In this regard, Song et al. (51) found that 12 weeks of ET reduced the extent of apoptotic DNA fragmentation in white gastrocnemius and soleus muscles of old rats, which was attributed to downregulation of mitochondrial apoptotic signaling. Similar findings were reported by Marzetti et al. (52) in old rats following 4 weeks of treadmill running. Notably, ET prevented the age-related elevation of TNF-α-related apoptotic signaling in the extensor digitorum longus muscle of old rats, which was associated with improved exercise capacity and muscle strength.

A potential role for ET in increasing the circulating levels of IGF-1 has also been suggested (38). For instance, 8 weeks of ET significantly increased fasting levels of IGF-1, especially in older men relative to women (49). There was also a significant correlation between changes in VO_2max_ and IGF-1 in men (49). In addition, basal levels of growth hormone (GH), IGF-1, and IGF binding protein 1 (IGFBP-1) were found to be higher in trained middle-aged men relative to sedentary controls (46). Furthermore, acute ET increased the activity of the GH/IGF-1 axis in middle-aged men (46). In support of the anabolic effect of ET, 3 h of bycicle exercise induced a sevenfold increase in plasma levels of follistatin as opposed to a smaller effect of one-legged knee extensor exercise (47). The increase in plasma follistatin after ET may be dependent on several factors, including the intensity and duration of exercise (47). In line with this, Sakamoto et al. (13) found that Akt activity significantly increased following acute submaximal and maximal intensity ET. Increases in Akt activity were accompanied by enhanced Akt Thr308 and Ser473 phosphorylation (13). The beneficial effects of ET on anabolic pathway may depend on the frequency of training. In support to this notion, Pasini et al. (59) investigated the effects of 8 weeks of ET and training frequency [i.e., 3 (EX3) or 5 days/week (EX5)] on anabolic pathways in skeletal muscle of old rats. Aging was associated with reduced protein levels of IRS-1 and p-mTOR in control rats relative to the young control group. In response to ET, EX3 resulted in reduced insulin receptor expression and increased IRS-1 levels compared with old sedentary rats. EX5 upregulated not only IRS-1 and COX activity but also p-mTOR expression (59).

### Resistance Training

RT is effective in preventing and treating sarcopenia due to its ability to promote net muscle protein anabolism, resulting in specific metabolic and morphological muscular adaptations (27, 66, 67).

The effects of acute and chronic RT on skeletal muscle in advanced age have been thoroughly investigated (10, 26, 31, 68–70). Nevertheless, the acute effects of RT are yet to be elucidated. Fry et al. (10) measured intracellular mediators of muscle protein synthesis (MPS) following an acute bout of RT in young and old persons. At baseline and 3, 6, and 24 h after RT, muscle biopsies were taken from the vastus lateralis. No changes were observed in phosphorylation of several key signaling proteins, mTOR, S6K1, 4E-BP1, and ERK1/2 following exercise in the older group. Increased MPS factors following exercise was found only in the younger group (10). On the other hand, Raue et al. (68) investigated the mRNA expression of several myogenic modulators at rest and 4 h after a single bout of RT in young and old women. Participants performed 3 sets of 10 repetitions of bilateral knee extensions at 70% of one-repetition maximum. RT led to upregulation of MyoD (2.0-fold) and MRF4 (1.4-fold) and downregulation of myostatin (2.2-fold) (68). The same group also investigated the effect of an acute bout of RT consisting of 3 sets of 10 knee extensions at 70% of one-repetition maximum on mRNA expression of ubiquitin proteasome-related genes involved in muscle atrophy in very old women (31). Muscle biopsies were taken from the vastus lateralis before RT and 4 h after. The authors demonstrated upregulation of atrogin-1 and MuRF-1 gene expression in response to RT. These data suggest that the regulation of ubiquitin proteasome-related genes involved in muscle atrophy is altered in very old women in response to RT (31).

In contrast to acute RT, Melov et al. (69) compared the expression profile of genes related to muscle strength, in healthy young and old men and women before and after a 6-month RT program. In response to RT, a significant improvement in strength was found in both age groups. Following RT, the transcriptional signature of aging was significantly reversed toward a youthful profile for most genes. The authors concluded that mitochondrial function and muscle weakness were favorably altered at the phenotypic and transcriptome level, following 6 months of RT (69). In support to the effects of RT on age-related changes in mitochondrial function, Luo et al. (70) investigated the signaling pathways that regulate autophagy and apoptosis in the gastrocnemius muscles of 18- to 20-month-old rats in response to 9 weeks of RT. Their findings demonstrated that RT prevented the loss of muscle mass which was accompanied by reduced microtubule-associated protein 1A/1B-light chain 3 (LC3)-II/LC3-I ratio, reduced p62 protein levels, and increased levels of autophagy regulatory proteins (Atgs), including Beclin 1, Atg5/12, Atg7, and the lysosomal enzyme cathepsin L. These improvements in autophagic signaling were associated with upregulation of total and phosphorylated AMPK and FoxO3A expression. Their results also showed that RT reduced cytochrome *c* release in the cytosol and caspase-3 activation suggesting an inhibition of apoptosis. Moreover, RT upregulated the expression of IGF-1 and its receptors and downregulated the phosphorylation of Akt and mTOR. As a whole, these findings suggest an anti-apoptotic effect of chronic RT most likely *via* inhibition of mitochondria-mediated apoptosis in aged skeletal muscle (70).

The exact mechanisms by which RT stimulates protein synthesis in old muscles are not yet fully understood. However, it has been speculated that in response to RT, IGF-1 and its receptors, as well as the Akt/mTOR and Akt/FoxO3a signaling pathways may be modulated (70). In fact, in response to RT, IGF-1 activates PI3K, which leads to membrane translocation and subsequent phosphorylation of Akt by phosphoinositide-dependent kinase (PDPK) 1 and 2. Once activated, Akt phosphorylates mTOR and glycogen synthase kinase 3 beta (GSK3B), which play an important role in protein synthesis, transcriptional and proliferative processes related to the hypertrophic response, and control of protein degradation (16). Other mechanisms that are involved in MPS are those regulating MAPK signaling. It has been shown that in response to RT, phosphorylation of ERK1/2 by MAPK is increased, and mTOR is activated (44). mTOR activation by the ERK pathway may occur through the phosphorylation of tuberous sclerosis complex 2 (TSC2) (16).

## Conclusion

Sarcopenia is a major corollary of aging. Most intracellular signaling pathways involved in muscle homeostasis are affected and could therefore be exploited as targets for the development of interventions aimed at preventing, delaying, or reversing sarcopenia. To date, physical exercise, especially if in combination with appropriate nutritional supplementation (71), is considered to be the only effective intervention to manage sarcopenia and prevent its adverse outcomes (72). As for the training modality, RT is more effective in increasing muscle mass and strength, whereas ET is superior for improving maximum aerobic capacity. Based on this evidence, older people should be recommended engaging in a balanced program of both endurance and strength exercises, performed on a regular schedule (73). Table 1 summarizes general guidelines for resistance and aerobic exercises for older adult based on the American College of Sports Medicine in conjunction with the American Heart Association recommendations (25). Future research aimed at addressing the simultaneous effects of endurance and resistance exercise along with drug and nutritional interventions would help clarify the pathogenesis of sarcopenia and its signaling pathways.

**Table 1:** American College of Sports Medicine/American Heart Association resistance and aerobic exercise recommendations for older adults.

	Frequency	Intensity	Duration	Type
Aerobic exercise	Moderate-intensity activities, accumulate at least 30 or up to 60 (for greater benefit) min/day in bouts of at least 10 min each to total 150–300 min/week, at least 20–30 min/day or more of vigorous-intensity activities to total 75–150 min/week	On a scale of 0–10 for RPE, 5–6 for moderate-intensity, and 7–8 for vigorous intensity	Moderate-intensity activities, accumulate at least 30 min/day in bouts of at least 10 min each or at least 20 min/day of continuous activity for vigorous-intensity activities	Any modality that does not impose excessive orthopedic stress; walking is the most common type of activity. Aquatic exercise and stationary cycle exercise may be advantageous for those with limited tolerance for weight-bearing activity
Resistance exercise	At least 2 days/week	Between moderate- (5–6) and vigorous- (7–8) intensity on a scale of 0–10	It may vary, depends on number of movements, sets, and repetitions	Progressive weight training program or weight-bearing calisthenics (8–10 exercises involving major muscle groups of 8–12 repetitions each), stair climbing, and other strengthening activities that use major muscle groups

*RPE, Borg rating of perceived exertion*.

## Author Contributions

MZ, together with EM, conceived the paper, conducted, supported, and interpreted results of literature review, and drafted the manuscript. AP assisted with the conception of the paper, contributed to the discussion, and assisted with editing the manuscript. ZM assisted with the literature review and manuscript drafting. All authors critically revised the manuscript and approved the final version.

## Conflict of Interest Statement

The authors declare that the research was conducted in the absence of any commercial or financial relationships that could be construed as a potential conflict of interest.
